# Real-time virtual sonography for prone-position breast MRI: technical feasibility and accuracy in locating 125 breast lesions

**DOI:** 10.1186/s41747-026-00726-x

**Published:** 2026-05-22

**Authors:** Leonhard Gruber, Michael Swoboda, Johannes Deeg, Birgit Amort, Silke Haushammer, Daniel Egle, Claudia Iannetti, Valentin K. Ladenhauf, Malik Galijasevic, Pietro Lacaita, Martin Daniaux, Benjamin Henninger, Elke R. Gizewski

**Affiliations:** 1 Department of Radiology, Medical University Innsbruck, Anichstraße 35, 6020 Innsbruck, Austria; 2 Department of Gynecology and Obstetrics, Medical University Innsbruck, Anichstraße 35, 6020 Innsbruck, Austria; 3 Department of Visceral, Transplant, and Thoracic Surgery (VTT), Centre of Operative Medicine, Medical University Innsbruck, Anichstraße 35, 6020 Innsbruck, Austria

**Keywords:** Breast, Image processing (computer-assisted), Magnetic resonance imaging, Prone position, Ultrasonography (interventional)

## Abstract

**Objective:**

This study evaluates the feasibility and technical success rate of real-time virtual sonography (RVS) for prone contrast-enhanced breast MRI sequences during second-look examinations. Usually, additional supine MRI sequences are acquired for coregistration.

**Materials and methods:**

This single-center retrospective study was performed in a cohort of female patients who underwent contrast-enhanced prone breast MRI followed by second-look ultrasound for MRI-detected incidental lesions. RVS was used to coregister supine ultrasound and prone MRI data without requiring additional supine MRI studies. Lesion localization success, as well as lesion visibility, fusion quality, and histopathological correlation through ultrasound-guided biopsy, were assessed. A covariate analysis of factors affecting lesion localization was performed.

**Results:**

A total of 103 female patients (mean age 48.3 ± 11.0 years) with 125 MRI-detected breast lesions were included. Of the lesions, 91.2% were successfully localized using RVS, including a high proportion of non-mass enhancements (41.6%). Ultrasound-guided biopsy was performed in 57.6% of cases, confirming malignancy in 31.9% of those. Covariate analysis identified higher breast volume as the only factor significantly associated with reduced RVS coregistration success (odds ratio 0.993, *p* = 0.035).

**Conclusion:**

RVS represents an advanced imaging approach in breast diagnostics, offering a promising solution to overcome the limitations of standalone modalities and potentially enhance diagnostic accuracy. We showed that prone MRI studies may be sufficient for RVS-based coregistration of breast lesions, potentially rendering additional supine MRI acquisitions unnecessary.

**Relevance statement:**

This study demonstrates that real-time virtual sonography with contrast-enhanced MRI in the prone position is a feasible and effective method for localizing MRI-detected breast lesions on second-look ultrasound without the need for additional supine MRI. This approach can optimize diagnostic workflows and reduce imaging burden while maintaining high localization rates.

**Key Points:**

- RVS localizes MRI-detected breast lesions in the prone position without requiring additional supine MRI for co-registration.
- Successful localization was achieved in 91.2%, including 41.6% non-mass enhancements, with higher breast volume as the only detrimental factor.
- RVS enables accurate lesion localization without supine MRI, streamlining workflows, lowering imaging costs, and improving biopsy access.

**Graphical Abstract:**

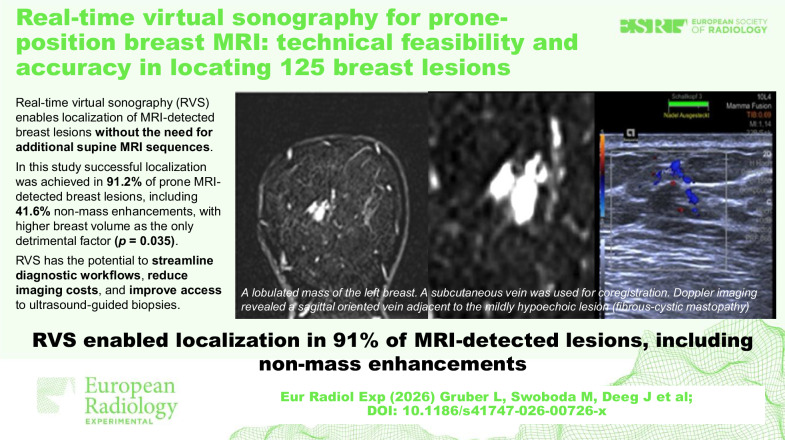

## Background

Breast cancer remains a significant global health concern with 2.3 million annual new cases and 670,000 deaths in 2022, with both counts being expected to rise in the future [1], necessitating continuous advancements in diagnostic imaging to enhance early detection and improve patient outcomes [2]. Apart from conventional mammography and tomosynthesis, sonography and magnetic resonance imaging (MRI) are two widely employed modalities in breast imaging, each offering unique advantages: MRI provides excellent soft tissue contrast and is highly sensitive in detecting breast lesions, providing a sensitivity range of 94% to 100% [3–5], while ultrasound excels in real-time imaging and cost-effectiveness [6].

Suspicious breast MRI-detected lesions require biopsy for histopathologic confirmation. While MRI-guided biopsy is safe and accurate, its use is limited by cost, availability, and the need for contrast administration and increased difficulty with lesions near the chest wall or nipple [7–9]. Consequently, in numerous settings, the classification of MRI-detected lesions often relies on second-look sonography, followed by biopsy. The effectiveness of a cognitive approach depends on the operator’s experience and shows very heterogenous detection rates [10]. This situation can lead to off-target biopsies and increased healthcare costs [11, 12].

To address this limitation, researchers have focused on utilizing MRI imaging via real-time virtual sonography (RVS) imaging to improve diagnostic precision [13]. Ultrasound-MRI fusion techniques involve the registration of real-time ultrasound images with pre-acquired MRI, allowing for a comprehensive evaluation of lesions in both spatial and functional dimensions. A common RVS approach uses an electromagnetic tracking system, where a sensor is attached to the ultrasound transducer, allowing for the accurate spatial co-registration of ultrasound and MRI images [14]. Combining the strengths of these modalities through ultrasound-based MRI fusion examination techniques has emerged as a promising approach to improve diagnostic accuracy and guide treatment decisions in breast cancer management [13].

Usually, patients undergo an additional supine breast MRI to generate a dataset comparable to the ultrasound setting regarding breast position and deformation before RVS. Conducting two MRIs presents a significant challenge for some institutions, as patients may have to undergo another MRI examination and receive additional contrast agent [13]. Therefore, to address this challenge the objective of this study was to retrospectively assess the feasibility and technical success rates of a supine-prone RVS approach based on a fusion of supine ultrasound and prone contrast-enhanced MRI without the need for another supine MRI acquisition.

## Materials and methods

### Study design

**Fig. 1:**
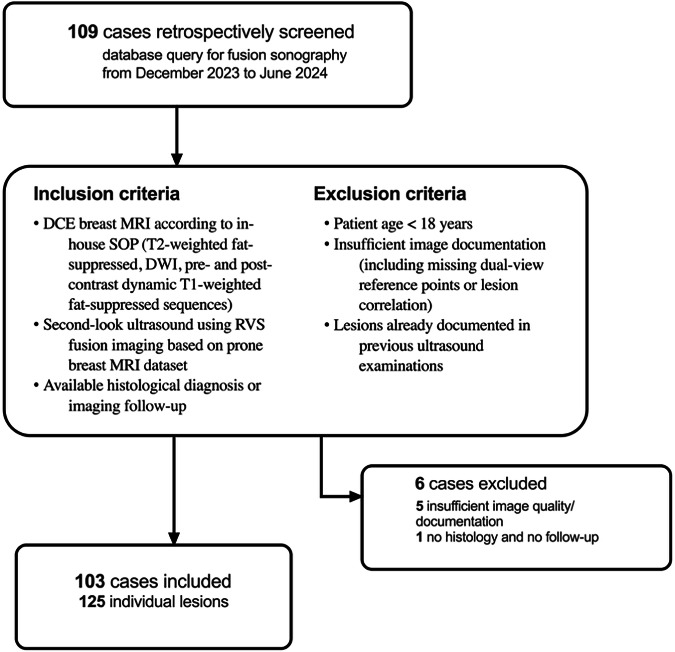
Flow chart of case screening numbers, inclusion and exclusion criteria, and included and excluded cases

This study was conducted in compliance with the principles outlined in the 2024 revision of the Declaration of Helsinki and received approval from the local Ethical Review Board on September 27, 2024. This single-center retrospective study involved 103 consecutive female patients who had undergone dynamic contrast-enhanced breast MRI in the prone position with RVS-based second-look ultrasound to assess incidental lesions initially detected on standardized MRI of the breast without correlation in prior ultrasound or mammography examinations between December 2023 and June 2024. Inclusion and exclusion criteria are reported in Table 1. Figure 1 depicts screening, inclusion and exclusion criteria and included case numbers.

**Table 1:** Inclusion and exclusion criteria

Inclusion criteria	Exclusion criteria
• Dynamic-contrast enhanced breast MRI in prone position following in-house SOP, including T2-weighted fat-suppressed, diffusion-weighted and pre- and post-contrast dynamic T1-weighted fat-suppressed sequences. • Second-look sonography using Real-time virtual sonography (RVS) fusion imaging of one or more lesions newly identified on breast MRI via a prone breast MRI dataset. • Available histological diagnosis or follow-up examinations, if no biopsy was performed.	• Patient age under 18. • Insufficient image documentation, including dual-view reference points and lesion correlation. • Lesions in question were already documented in previous ultrasound examinations.

### Contrast-enhanced breast MRI examination in the prone position

MRI scans were conducted using a MAGNETOM 1.5-T and 3-T scanners (AvantoFit and Skyra, Siemens Healthineers) using an 18-channel breast coil (Siemens Healthineers). All patients underwent a prone position MRI following an in-house protocol. The MRI scan protocol encompassed diffusion-weighted imaging, T2-weighted TIRM, and both an unenhanced and contrast-enhanced axial T1-weighted fast low-angle shot three-dimensional sequence (dynamic post-contrast images at 4 time points). Sequence details are provided in supplementary Table S1. Gadobutrol 1 mmol/mL (Gadovist; Bayer AG) at a dose of 0.1 ml/kg body weight was administered intravenously at a rate of 2.0 mL/s, followed by a 20-mL saline flush

Unenhanced images were subtracted from the corresponding contrast-enhanced images on a pixel-by-pixel basis, and maximum intensity projections were generated from these subtraction images. All MRI examinations were evaluated by radiologists specialized in breast imaging: L.G. (7 years of breast-imaging experience), A.B. (15 years of breast-imaging expertise) or S.H. (10 years of breast-imaging expertise). MRI studies are usually interpreted by a primary reader with potential consultation of another team member in unclear or challenging cases. Any suspicious enhancing lesion was evaluated based on its shape, borders, distribution, internal architecture, and kinetic analysis, following the BI-RADS Atlas [15]. The precise location within the breast was assessed based on the shortest distance to the pectoral muscle (PM), skin, and nipple. Contrast-enhancement strength was visually compared to PM uptake on subtraction images as none (no signal), mild (faint uptake, below PM level), moderate (intermediate uptake, at PM level) and strong (high uptake, above PM level). Additionally, breast volume, amount of glandular tissue and background enhancement were assessed.

### Supine second-look examination with RVS

All sonographic examinations were conducted by either L.G., A.B. or S.H. The examiner did not have to be the initial reader of the MRI. The sonographic examination was performed with patients lying in the supine position with the affected arm raised. While routine scanning encompasses both the longitudinal and transverse planes, RVS for lesion detection was performed without conventional ultrasound, *i.e.*, the first step was landmark co-localization. The RVS-system consisted of a compact freehand sensor (General Purpose Electromagnetic Sensor, Civco) attached to the side of a 10L4 ultrasound transducer (Siemens Heathineers, 2.9–9.9 MHz linear-array probe) and an electromagnetic transmitter (Transmitter Mid Range, Siemens Heathineers) that remained fixed in a designated position (typically to the left of the patient’s head) to determine the relative position of the ultrasound probe within the transmitter’s defined operational field. For RVS purposes, the post-contrast phase T1-Flash3D volume data with the highest lesion-to-background ratio (by visual assessment) was used. Examiners were free to use the subtracted or non-subtracted sequence version. A standardized B-mode preset including harmonic imaging was used. Reference points were chosen depending on the lesion localization. Such landmarks had to be easily identifiable by both sonography and MRI and could include the nipple, vessels, other lesions, clips, or the chest wall. Correct co-registration was documented. To correct for (supine) breast mobility, the examiners were allowed to perform minor adjustments to the MRI images through rotation and translational movement in the *x*- and *y*-directions, but not along the *z*-axis, after co-registration. RVS fusion quality was assessed by M.S. (one year of breast-imaging expertise), blinded to the examiner, using a 5-point scale—1 (poor), 2 (fair), 3 (good), 4 (very good), and 5 (excellent)—based on the real-time alignment of the MRI and ultrasound images, as documented. Additionally, lesion’s visibility on ultrasound was evaluated by an experienced operator (L.G. (7 years of experience), A.B. (15 years) or S.H. (10 years)) and classified as barely, moderately, or well discernible.

### Biopsy procedure and histopathological correlation

Breast biopsies were carried out following a standardized operating procedure according to international guidelines [16] after detailed patient information and acquisition of a signed informed consent. A small skin incision was made after skin disinfection and intracutaneous and ultrasound-guided perilesional application of Mepivacaine hydrochloride 1%. Under constant ultrasound-visualization, up to five tissue specimens are acquired using a 12 G or 14 G core-needle biopsy system (HistoCore Automatic Biopsy System; BIP GmbH). Core-needle specimens were embedded in a 5%-formalin solution for further staining and analysis. For patients who did not undergo a biopsy but were classified as BI-RADS 3 lesions, a follow-up examination (according to BI-RADS guidelines) was conducted [15]. All biopsy sites were routinely marked by placing either an UltraClip Dual Trigger Biodur 108 Coil Marker (Becton Dickinson), TUMARK Professional (Somatex Medical Technologies), TUMARK Vision (Somatex Medical Technologies), or HydroMARK Breast Biopsy Site Marker (Mammotome), depending on lesion localization and potential allergies to marker components.

### Statistical analysis

All data were stored in Microsoft Excel 16.93.1 (Microsoft). Statistical software used was SPSS 27.0 for Windows (SPSS) and GraphPad Prism 10.4.1 (GraphPad Software LLC). Statistical significance was considered for *p*-values < 0.05.

Descriptive statistics for all patients include demographic (age, familial history of breast cancer, prior breast cancer) and disease-related factors, frequency of benign, intermediate and malignant tumors (including subtypes). A binary regression analysis for technical success was carried out after exclusion of factor collinearity via variance-inflation factor statistics. Results include regression factor B, standard error, odds ratio, and *p*-value.

## Results

### Patient demographics

Overall, 103 female patients aged 48.3 ± 11.0 years (mean ± standard deviation), range 22.5–73.4 years, with 125 MRI-detected lesions were included in this study. In 81 cases (78.6%), the breast MRI was performed due to a familial or established genetic high-risk situation, the rest (*n* = 22, 21.4%) due to a newly diagnosed breast tumor or symptoms such as nipple secretion, pain or equivocal ultrasound findings. In detail, patients had been referred to MRI for high-risk screening in 28 cases (27.2%), symptoms without correlate in ultrasound or mammography in 8 cases (7.8%), pre-operative staging in 16 cases (15.5%), follow-up or assessment of BI-RADS 3 lesions in ultrasound or mammography in 33 cases (32.0%), sonographically occult findings (BI-RADS 0) in 5 cases (4.9%), and unclear reasons in 13 cases (12.6%). The most common breast glandular distribution on MRI was heterogeneous fibroglandular tissue (40.8%) and mild background parenchymal enhancement (36%) (Table 2).

**Table 2:** Clinical and MRI features of included patients

Parameter	
Sex (*n*, %)	
Female	103 (100.0)
Age group (*n*, %)	
18–39	27 (21.6)
40‒59	84 (67.2)
> 60	14 (11.2)
Indications for MRI (*n*, %)	
Family history of breast cancer/genetic predisposition	28 (27.2)
Symptoms without US correlate	8 (7.8)
Newly diagnosed breast cancer with preoperative staging	16 (15.5)
Follow-up examination	33 (32.0)
Mammographic finding without US correlate (BI-RADS 0)	5 (4.9)
Unknown	13 (12.6)
Implant (yes) (*n*, %)	8 (6.4)
Breast volume (approx.) (cm^3^)	221.0 ± 135.5 (range 15.6 to 679.2)
Breast glandular distribution (*n*, %)	
Almost entirely fatty	12 (9.6)
Scattered fibroglandular tissue	25 (20.0)
Heterogeneous fibroglandular tissue	51 (40.8)
Extremely dense fibroglandular tissue	37 (29.6)
Background parenchymal enhancement (*n*, %)	
Minimal	38 (30.4)
Mild	45 (36.0)
Moderate	30 (24.0)
Marked	12 (9.6)

### Characteristics of breast lesions detected on MRI

Among the 125 individual breast lesions, 54.4% were located in the right breast, with the lower outer quadrant accounting for the most cases at 34.4%. Of note, in a high percentage (41.6%) of cases, non-mass enhancements were RVS-targeted. The mean lesion size on MRI showed a high spread due to mass and non-mass targets (13.8 ± 10.7 mm, range 3 to 67 mm). On average, mass-like lesions (*n* = 74) measured 8.7 ± 4.9 mm (range 3 to 34 mm), while non-mass-like lesions (*n* = 51) reached 21.0 ± 12.3 mm in maximum diameter (range 5 to 67 mm). Table 3 provides an overview of the MRI characteristics of the breast and the respective lesion.

**Table 3:** MRI characteristics of detected breast lesions (*n* = 125)

Lesion type (*n*, %)	
Mass	74 (58.4)
Non-mass enhancement	51 (41.6)
Quadrant (*n*, %)	
Upper outer	36 (28.8)
Lower outer	43 (34.4)
Lower inner	22 (17.6)
Upper inner	24 (19.2)
Breast side (*n*, %)	
Right	57 (45.6)
Left	68 (54.4)
Perilesional breast tissue (*n*, %)	
Almost entirely fat	15 (12.0)
Mixed	85 (68.0)
Mostly glandular	25 (20.0)
Max. lesion diameter (mm)	13.8 ± 10.7 (range 3 to 67)
Lesion volume (cm^3^)	0.9 ± 2.0 (range 0.01 to 17.3)
Shortest distance to skin (mm)	17.9 ± 10.2 (range 1 to 46)
Shortest distance to pectoral muscle (mm)	38.6 ± 29.3 (range 1 to 116)
Shortest distance to nipple (mm)	45.8 ± 22.6 (range 1 to 110)
Mass shape (*n*, %)	
Oval	26 (35.1)
Round	5 (6.8)
Irregular	43 (58.1)
Mass margin (*n*, %)	
Circumscribed	38 (51.4)
Non-circumscribed, spiculated	23 (31.1)
Non-circumscribed, irregular	13 (17.6)
Contrast enhancement strength (*n*, %)^a^	
None	0 (0.0)
Mild	11 (8.8)
Moderate	47 (37.6)
Strong	67 (53.6)
Kinetic curve assessment (*n*, %)	
I	43 (34.7)
II	43 (34.7)
III	38 (30.6)
Apparent diffusion coefficient value	1365.2 ± 302.2 (range 592 to 2381)
Lesion BI-RADS score	
3	53 (42.4)
4	71 (56.8)
5	1 (0.8)
Overall BI-RADS score (MRI + RVS)^a^	
3	43 (34.4)
4	67 (53.6)
5	7 (5.6)
6	8 (6.4)

^a^ Reflects combined lesion grading in MRI and RVS, as well as presence of other histologically confirmed tumors (BI-RADS 6)

### Second-look evaluation using RVS

**Fig. 2:**
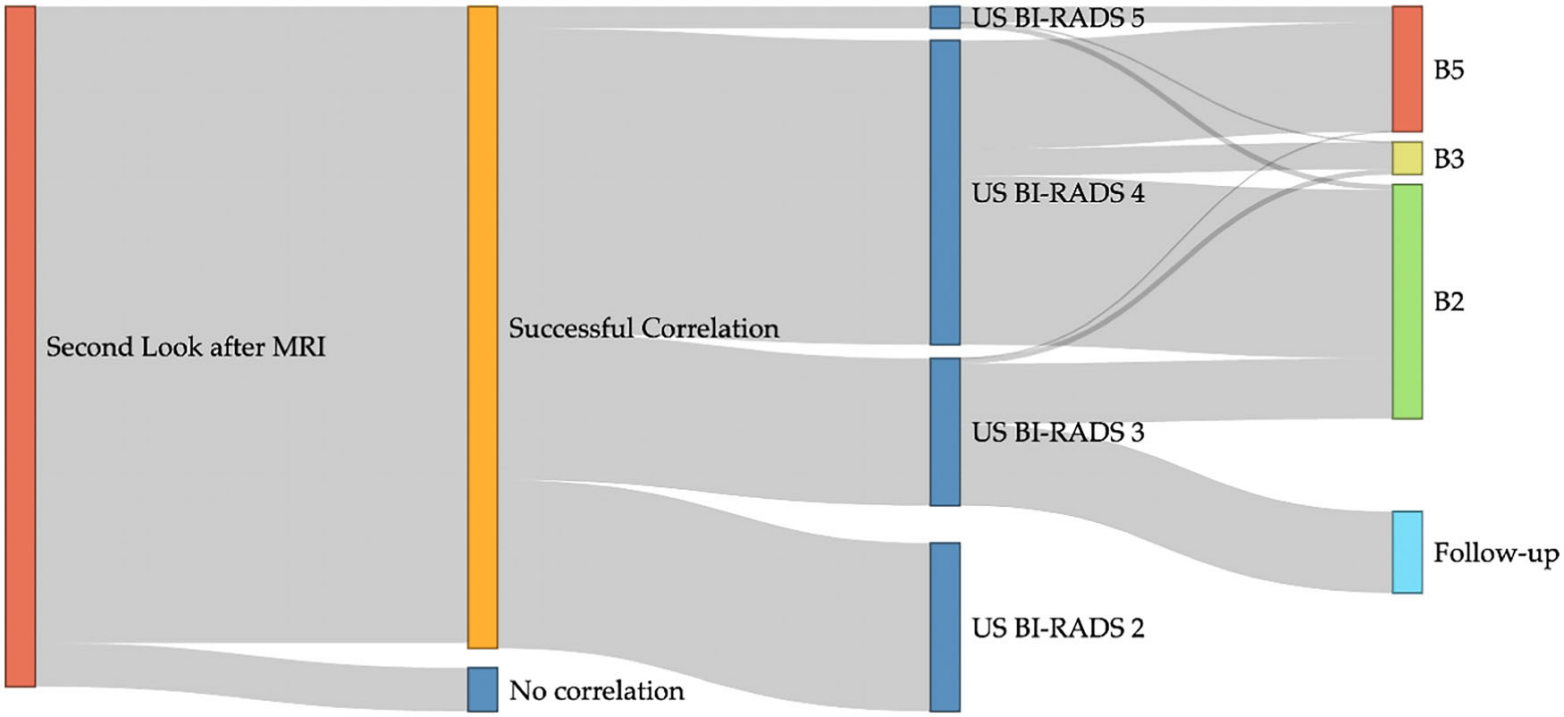
Diagnostic flow of patients undergoing second-look evaluation with real-time virtual ultrasound, including BI-RADS classification and final outcomes

Among the 125 individual breast lesions, 114 (91.2%) could be localized by RVS. Figure 2 shows the diagnostic pathway from MRI second-look evaluation with RVS-correlation to BI-RADS classification and final outcomes, including histopathological results. On average, 1.6 ± 0.6 reference points were used, with the nipple or chest wall/implant surface serving as the most common points of reference. Overall, 57.6% of cases (*n* = 72) underwent ultrasound-guided biopsy. Of those lesions, histopathological evaluation revealed 31.9% (*n* = 23) to be malignant, 8.3% (*n* = 6) intermediate and 59.7% (*n* = 43) benign. The most common benign histopathological condition was fibrocystic mastopathy (72.1%, *n* = 31), while the most frequent malignant finding was invasive carcinoma of no special type (39.1%, *n* = 9). All biopsied lesions were marked with a clip.

Of the lesions that were successfully co-registered and underwent follow-up rather than biopsy due to typical benign presentation, 46.1% (*n* = 24) were classified as fibrous-cystic mastopathy, 13.5% (*n* = 7) as fibroadenoma, 1.9% (*n* = 1) as glandular tissue, 1.9% (*n* = 1) as inflammatory, 1.9% (*n* = 1) as scar tissue, 9.6% (*n* = 5) as typically cystic, and 25.0% (*n* = 13) as other. Table 4 provides a more detailed overview.

**Table 4:** Parameters of real-time virtual sonography with histopathological results

Parameter	
Localization (yes) (*n*, %)	114 (91.2)
Fusion quality (1‒5)^a^	3.5 ± 1.0
Quality score 1 (*n*, %)	5 (4.0%)
Quality score 2 (*n*, %)	12 (9.6%)
Quality score 3 (*n*, %)	40 (32.0%)
Quality score 4 (*n*, %)	50 (40.0%)
Quality score 5 (*n*, %)	18 (14.4%)
Points of reference (*n*)	1.6 ± 0.6
Point of reference, type^b^	
Nipple	64 (31.7)
Chest wall/implant surface	53 (26.2)
Known other histologically verified lesion	28 (13.9)
Breast parenchyma	15 (7.4)
Cyst	27 (13.4)
Vessel	15 (7.4)
Visibility of the lesion	
Poor	10 (8.0)
Moderate	52 (41.6)
Good	63 (50.4)
Skin distance (mm)	6.5 ± 4.0
Pectoral distance (mm)	6.4 ± 5.7
Breast thickness (mm)	23.9 ± 11.4
Biopsy rate (*n*, %)	72 (57.6)
B2 lesions (*n*, %)	
Fibrous-cystic mastopathy	43 (59.7)
Fibroadenoma	31 (72.1)
Glandular tissue	1 (2.3)
Scar	6 (14.0)
Adenoma	3 (7.0)
Other	1 (2.3)
B3 lesions (*n*, %)	6 (8.3)
Papillomatous neoplasia	5 (83.3)
Atypical ductal hyperplasia	1 (16.7)
B5 lesions (*n*, %)	23 (31.9)
Invasive carcinoma, no special type (NST)	12 (52.2)
Ductal carcinoma *in situ*	10 (43.5)
Malignant other	1 (4.3)
Tumor grade	
G1	8 (34.8)
G2	10 (43.5)
G3	5 (21.7)

^a^ Higher is better

^b^ *n* higher than the overall case number due to multiple reference points in single cases

### Covariate analysis of factors affecting lesion localization

A covariate analysis revealed that only higher breast volume was associated with negative RVS correlation (odds ratio 0.993, *p* = 0.035), while other factors such as overall and perilesional glandular content, background parenchymal enhancement, lesion-to-skin and lesion-to-nipple distances, or lesion size had no significant impact (Table 5).

**Table 5:** Binary regression analysis of cofactors influencing technical RVS success

	Regression coefficient B	Standard error	*p*-value	Odds ratio
Breast volume (cm^3^)	-0.007	0.003	0.035	0.993
Breast glandular distribution (almost entirely fatty, scattered fibroglandular tissue, heterogeneous fibroglandular tissue, extremely dense fibroglandular tissue	-2.433	1.501	0.105	0.088
Background parenchymal enhancement (minimal, mild, moderate, marked)	-1.094	0.921	0.235	0.335
MRI lesion-to-skin distance (mm)	0.048	0.047	0.313	1.049
MRI lesion-to-nipple distance (mm)	-0.009	0.021	0.651	0.991
Points of reference (*n*)	0.332	0.717	0.643	1.394

## Discussion

**Fig. 3:**
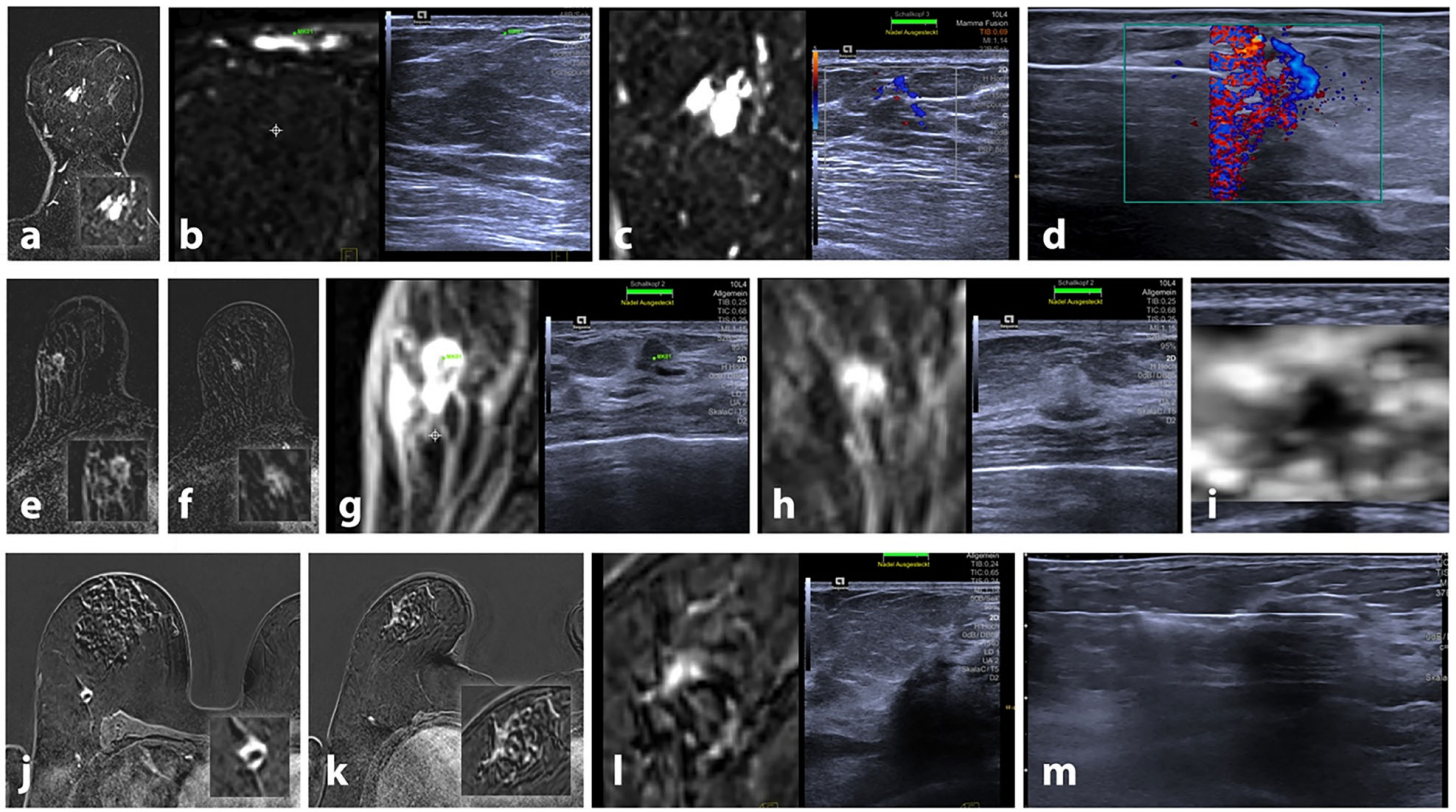
Three exemplary cases of successful RVS lesion localization. Case 1 (upper row): breast MRI revealed a small lobulated mass of the left breast in an approximately 35-year-old female patient (**a**). A subcutaneous vein was used for co-registration (**b**; green dots). Doppler imaging revealed a characteristic sagittal-oriented vein adjacent to the mildly hypoechoic lesion (**c**), which was confirmed to be fibrous-cystic mastopathy (**d**). Case 2 (middle row): in an approximately 60-year old female patient with a recently diagnosed invasive carcinoma of the right breast (**e**), a secondary lesion not appreciated during initial staging ultrasound was found upon staging MRI (**f**). After coregistration of the known tumor (**g**; green dots), a corresponding hyperechoic area (**h**) exhibiting elastographic hardening (**i**) was localized and histologically confirmed to be an invasive breast carcinoma, no special type G2. Case 3 (lower row): similarly, in an approximately 75-year old female patient with a newly diagnosed invasive carcinoma of the right breast (**j**) an ipsilateral non-mass enhancement was found in the staging MRI (**k**). Real-time virtual sonography revealed a corresponding hypoechoic area (**l**), which was confirmed to be an invasive breast carcinoma, no special type, G2 (**m**)

Contrast-enhanced breast MRI is known for its high sensitivity in detecting incidental lesions that often require further diagnostic evaluation. In this study, we analyzed 125 breast lesions identified on MRI and assessed the technical feasibility of RVS for localizing these lesions without the need for additional supine-position breast MRI. RVS successfully detected 114 of the 125 lesions, yielding a detection rate of 91.2%. In 57.6% of all cases, a histopathological confirmation through ultrasound-guided biopsy was performed. Covariate analysis indicated that higher breast volume was the only factor significantly associated with insufficient RVS correlation (odds ratio 0.993, *p* = 0.035). In contrast, variables such as overall and perilesional glandular tissue content, background parenchymal enhancement, lesion-to-skin and lesion-to-nipple distances, as well as lesion size, showed no significant impact on RVS performance. Exemplary cases of successful RVS are presented in Fig. 3.

In many studies, lesions initially identified on prone MRI but remaining occult during second-look ultrasound prompted the use of an additional supine MRI for RVS fusion guidance [17–19]. This approach addresses the notable discrepancy caused by breast deformation due to positional differences between prone and supine imaging [20]. Previous research by Carbonaro et al demonstrated that prone MRI can lead to lesion displacement of approximately 3–6 cm across the three orthogonal planes [21]. However, acquiring an additional supine MRI is both resource-intensive and time-consuming, limiting its practicality in routine clinical workflows [8]. In our study, we demonstrated a high lesion detection rate using the RVS fusion technique with a prone MRI dataset, with higher breast volume emerging as the only factor significantly associated with reduced fusion accuracy. This may be attributed to increased tissue deformation and greater variability in lesion position between the prone MRI and supine ultrasound examinations in larger breasts, making precise anatomical correlation more challenging. Notably, we achieved a high detection rate even in the context of relatively small lesions, with a mean size of 8.7 ± 4.9 mm for solid lesions in our study cohort, highlighting the sensitivity of RVS.

The technical success of ultrasound-MRI fusion techniques is a critical factor in determining their clinical utility. Studies assessing the accuracy and reliability of these fusion methods have reported promising results [22]. Some studies, such as that by Park et al, have demonstrated the feasibility of fusion imaging using only prone MRI datasets, reporting detection rates exceeding 90%. However, these findings are based on relatively small patient cohorts, ranging from 33 to 67 individuals [14, 23, 24]. In our study with over 100 patients, we also observed a high proportion of non-mass enhancement (NME) lesions (41.6%), which are often challenging to detect using conventional ultrasound due to their subtle and diffuse appearance [25]. These lesions, however, are typically well-visualized on contrast-enhanced MRI [26]. The use of the RVS fusion technique proved valuable in our study, enabling real-time localization and targeted ultrasound-guided biopsy of lesions that would otherwise be difficult to sample.

Our conventional approach partially relies on cognitive fusion to identify lesions that would otherwise remain occult on conventional ultrasound. As previously described, this technique requires a keen awareness of identifiable anatomical landmarks, such as adjacent vessels, cysts, and distinct tissue features like parenchymal islands or Cooper’s ligaments. In our experience, the choice of reference points for image co-registration depended on lesion location—for example, lesions near the thoracic wall required deeper anatomical landmarks. Consequently, a certain level of examiner experience remains essential for accurate lesion localization and successful fusion. Unsubtracted MRI sequences can aid in achieving better anatomical correlation during co-registration, as they provide improved visibility of anatomical landmarks, yet may be hampered by any significant amount of benign parenchymal enhancement obfuscating other landmarks. As this was a feasibility study, a certain proportion of lesions might have been successfully localized by using ultrasound alone. This was not the goal of this study, though.

The primary goal of our approach with RVS was to increase efficiency and minimize patient discomfort: Firstly, by reducing the number of MRI biopsies, which can be stressful and uncomfortable for patients as well as time- and cost-intensive for the healthcare system [27]. Secondly, by avoiding the acquisition of another prone position breast MRI in patients with incidental MRI findings—lesions that had not been identified on prior mammography or ultrasound—warranting second-look evaluation and eventually biopsy, time to diagnosis can be significantly shortened. Furthermore, patients with lesions not accessible to MRI biopsy due to breast size or lesion localization can also benefit from an RVS-guided biopsy. As previously noted, ultrasound identification was feasible for a range of lesions that were initially localized using RVS. It should be noted that in ultrasonography, visual interpretation of a tissue feature can be vastly different between *a priori* and *a posteriori* situations [13, 28].

Advancements in machine learning algorithms have contributed to the refinement of fusion techniques [28, 29]. Artificial intelligence-based systems, such as deep learning models, have shown promise in automating the registration process and improving the overall accuracy of ultrasound-MRI fusion imaging [30]. Our method now does not appear to be a primary application for artificial intelligence-based systems, as the co-registration process relies on a very dynamic approach combining ultrasound and MRI experience with thorough knowledge of breast anatomy to compensate for the inherent supine-prone incongruency of ultrasound and MRI information due to a lack of any elastic transformation of the two volumes. Still, future AI-based co-registration might be able to dynamically transform the MRI dataset to fit the *in vivo* supine breast anatomy.

Additionally, not only MRI datasets but also CT images can be used for fusion. Chest computed tomography is routinely employed to assess lung diseases, and with its increased use, the incidental detection of breast lesions has become more common. Studies showed that targeted sonography using RVS is an effective method for localizing breast lesions incidentally detected on chest CT or ^8^F-FDG PET/CT [31, 32].

As this study focused on technical feasibility, the aspect of MRI-based breast imaging overdiagnosis could not be addressed [33]. Especially smaller lesions may never have been detected and remained clinically irrelevant, thus potentially leading to unnecessary follow-up examinations or biopsies. Nonetheless, biopsy was deemed necessary in over half of our cohort. Of those histologically confirmed lesions, a third were malignant, with the smallest solid lesion reaching 3 mm in maximum diameter, the smallest non-mass lesion 5 mm.

Ultrasound-based MRI fusion techniques offer a complementary approach in breast imaging, aiming to mitigate some limitations of the individual modalities and potentially enhance diagnostic accuracy. By examining the technical aspects and reported success rates of these methods, this integration appears to provide a useful addition to current breast cancer diagnostic tools. Furthermore, translation to CT/US fusion seems certainly feasible.

There are some limitations to this study. Firstly, an update to the BI-RADS lexicon has recently been published [34]. Several changes were introduced, touching, among others, on MRI breast density assessment and lesion descriptors. As this study was conceived and conducted before this update, details may differ. Still, the main aim of this study was to demonstrate the technical feasibility of prone-supine co-registration.

Secondly, in theory, a portion of the lesions identified in our study using RVS might have been localized using solely a cognitive fusion approach, with an examiner trying to identify an area of interest through non-technical recognition of landmarks and overall anatomy. To avoid co-registration bias, we skipped this step and immediately used RVS, even though the initial landmark choice may depend on the perceived lesion location in MRI. Interreader variability was not assessed, as fusion-based lesion localization was performed by one of three core readers (L.G., B.A., S.H.) without repeated identification, owing to the retrospective clinical design of the study. Future research should aim to evaluate inter-reader consistency in this context. It should be noted that not all breast lesions identified using RVS fusion imaging underwent biopsy, which limits the ability to fully correlate imaging findings with histopathological results.

Furthermore, RVS can be time-consuming, especially in patients with difficult parenchyma mimicking lesions of interest or very large or mobile breasts. Even though fusion co-registration quality was rated mostly fair to moderate, success rates were high. Fusion techniques can be particularly challenging in organs such as the breast, which lack fixed anatomy and size due to their non-stationary, deformable nature [13, 35]. This illustrates that a cognitive fusion component may still play an integral part in this setting. Overall, RVS is a highly specialized technique and may not be feasible to implement in daily practice due to constraints regarding time or resources and therefore may be most suited for tertiary centers. Lesions may appear unnaturally large when scaled to size by ultrasound-co-registration, which may hamper the orientation. Here, a cognitive component comes into play; additionally, viewing the MRI on a second screen may help. Due to cost considerations, no post-biopsy MRI examination was performed, which represents a corresponding limitation.

In conclusion, we demonstrated that ultrasound-MRI fusion using only prone MRI data—without the need for an additional supine MRI—achieves a high lesion detection rate (91.2%), including non-mass enhancements that are often difficult to identify with ultrasound alone. Higher breast volume was the only factor significantly associated with reduced fusion accuracy (*p* = 0.035). These results highlight the clinical feasibility and efficiency of RVS fusion imaging in breast diagnostics.

## Supplementary information

**Additional file: Table S1** Contrast-enhanced breast MRI sequence parameters.

## Data Availability

The datasets generated and analyzed during the current study are not publicly available but are available from the corresponding author on reasonable request.

## Abbreviations

PM: Pectoral muscle
RVS: Real-time virtual sonography
