# Supplementary material for: Real-time virtual sonography for prone-position breast MRI: technical feasibility and accuracy in locating 125 breast lesions
Source: Eur Radiol Exp. 2026 May 22;10:73. doi: 10.1186/s41747-026-00726-x (PMC13197569; doi:10.1186/s41747-026-00726-x)
Supplement: Supplementary file 1 — Additional file: Table S1 Contrast-enhanced breast MRI sequence parameters. [file 41747_2026_726_MOESM1_ESM.pdf]

Real-time virtual sonography for prone-position breast MRI: technical feasibility and accuracy in locating 125 breast lesions.

ELECTRONIC SUPPLEMENTARY MATERIAL

Supplementary material

Table S1 Contrast-enhanced breast MRI sequence parameters

	T2-TIRM	T1-FLASH 3D	RESOLVE-DWI
Repetition time (ms)	5,150	5.32	8,710
Echo time (ms)	61	1.93	61
Inversion time (ms)	@1.5 T: 170<br>@ 3 T: 230	-	-
Flip angle (°)	90	10	90
Turbo factor	11	-	5
Receive bandwidth (Hz/Px)	228	300	610
Fat suppression	STIR	SPAIR	SPAIR
Parallel imaging	GRAPPA	GRAPPA	GRAPPA
Acceleration factor	3	3	2
b-values (s/mm2)	-	-	50/400/800
Averages	2	1	1/3/5
Slice thickness (mm)	4	1.6	4
Number of slices	35	112	42
Slice orientation	Transversal	Transversal	Transversal
Field of view (mm x mm)	300 x 300	300 x 300	360 x 180
Acquisition matrix	448 x 336	480 x 422	216 x 98
Acquisition time (min:s)	2:41	1:27	4:23
